# Surgical Treatment for Patients with Krukenberg Tumor of Stomach Origin: Clinical Outcome and Prognostic Factors Analysis

**DOI:** 10.1371/journal.pone.0068227

**Published:** 2013-07-09

**Authors:** Wei Peng, Rui-Xi Hua, Rong Jiang, Chao Ren, Yong-Nin Jia, Jin Li, Wei-Jian Guo

**Affiliations:** 1 Department of Medical Oncology, Fudan University Shanghai Cancer Center, Fudan University, Shanghai, China; 2 Department of Gynecology, Ovarian Cancer Program, Fudan University Shanghai Cancer Center, Fudan University, Shanghai, China; 3 Department of Medical Oncology, SunYat-sen University Cancer Center, Guangzhou, Guangdong, China; 4 Department of Gastrointestinal Surgery, Beijing Cancer Hospital &Institute, Peking University School of Oncology, Beijing, China; 5 Department of Oncology, The First Affiliated Hospital, Sun Yat-Sen University, Guangzhou, Guangdong, China; 6 Department of Oncology, Shanghai Medical College, Fudan University, Shanghai, China; Cedars Sinai Medical Center, United States of America

## Abstract

Krukenberg tumor originated from stomach in female patients is common in clinical practice, but it is still uncertain whether surgical resection of ovarian metastases could improve the outcome. Some studies suggested that a certain group of patients could benefit from the resection of ovarian metastases. However, conclusions were different between studies and there was no data to illustrate if certain molecular markers were associated with patients’ survival. In this study, we analyzed the effects of resection of ovarian metastases, and investigated prognostic factors in 133 patients with ovarian metastases originated from stomach. Furthermore, we examined the expression of some cancer stem cells (CSCs) markers or related molecules in 64 ovarian metastases specimens and analyzed the correlation between these molecules and patients’ survival. We found that the median overall survival (mOS) of all 133 patients was 16 months, and “gastrectomy” and “without ascites” were two independent prognostic factors associated with longer survival. The mOS of the patients with gastrectomy was longer than that of patients had not undergone gastrectomy (19 vs. 9 months, *p = 0.048*). Patients without ascites survived longer than those with ascites (mOS: 21 vs. 13 months, *p = 0.008*). We also found that Sox2, CD44 or CD133 positive expression in ovarian metastases were risk factors correlated with poor survival, and Sox2 expression was an independent prognostic indicator. These results suggested that ovarian metastasectomy might help to prolong the survivor of some patients with Krukenberg tumor originated from stomach. Patients without ascites, and with resected or resectable primary gastric cancer lesion could get benefit from and be potential candidate for surgical treatment. The expression of Sox2 might serve as a prognostic indicator for predicting patients’ survival and be helpful for selecting patients in future.

## Introduction

Ovarian metastases originated from stomach (primarily defined as krukenberg tumor), is usually seen in female patients with gastric cancer [1], [2]. It is considered as a late stage disease and the prognosis is still very poor. Until now, optimal treatment has not been established, and it is still uncertain whether surgical resection of ovarian metastases could improve the outcome. Some studies have investigated the role of metastasectomy in the treatment of krukenberg tumor originated from stomach and demonstrated that resection of ovarian metastases might prolong survival [3]–[6]. However, the role of ovarian metastasectomy is still under debate and only a certain group of patients might benefit from resection of ovarian metastases. Some studies indicated that ovarian metastasectomy was beneficial when gross residual diseases were thoroughly eradicated [7]–[9], while some other studies suggested that metachronous ovarian metastases or unilateral ovarian metastases might correlate with good survival [10]–[13]. However, the samples of these studies were small (less than 70 cases) and some of these studies enrolled patients with other origins including colon cancer and breast cancer, but evaluated them all together without discrimination. Therefore, we exclusively and retrospectively analyzed the outcome of metastasectomy in more patients (n = 133) with krukenberg tumor originated from stomach to get more convincing evidence and investigated the clinical and pathological prognostic factors to help selecting subgroups of patients that might benefit from surgical treatment.

Apart from clinical and pathological factors, we also paid attention to molecular biomarkers that might influence the prognosis. Latest studies suggested that cancer stem cells (CSCs), which are highly tumorigenic and exist as a minority population within tumors, play significant roles in cancer initiation, progression and metastasis, and treatment failure [14], [15]. CD44 and CD133 are two stem cell biomarkers extensively investigated and employed to identify a series of CSCs including ovarian CSCs and gastric CSCs [14]–[18]. Besides CD44 and CD133, many molecules are also regarded as CSCs related molecules or biomarkers, such as Oct4, Sox2, p-AKT, p-ERK, Bmi-1, Mel-18 and CBX7. These molecules are involved in the maintenance of stemness, or related with tumorigenesis, progression and prognosis of some kinds of cancer [19]–[25]. So we proposed that some of these CSCs related molecules might correlate with the survival of patients who underwent resection of ovarian metastases originated from stomach. By investigating the clinical and pathological characteristics of the patients and the CSCs related molecules’ expression profile of the ovarian metastases, we aimed to identify risk factors of prognosis which might help to select patients that could benefit from the resection of ovarian metastases, consequently providing optimal treatment strategy for patients with ovarian metastases originated from stomach.

## Materials and Methods

### Patients

From March 1998 to March 2011, 133 patients who underwent resection of ovarian metastases originated from stomach were retrospectively reviewed. Among them, the primary lesion and ovarian metastases were both resected in 89 patients, while 44 patients underwent only ovarian metastasectomy. Among 69 patients with synchronous metastases, 25 patients underwent both gastrectomy and ovarian metastasectomy, and 44 patients only underwent ovarian metastasectomy. All the patients’ cancer specimens were reviewed by a board of pathologists and were confirmed as gastric carcinoma and krukenberg tumor originated from the stomach. The expression of CSCs related molecules was examined in ovarian metastatic specimens obtained from 64 patients from the total population. Patients with cancer of multiple origins and serum albumin<35 g/L before ovarian metasetasectomy were excluded.

### Identification of Clinical-pathological Characteristics

Clinical-pathological characteristics that possibly influence the prognosis of patients were identified and showed in Table 1, including age (>50 or ≤50 years), synchronous or metachronous ovarian metastases, gastrectomy(yes/no), extra-ovarian metastases before resection of ovarian metastases (yes/no), ascites before resection of ovarian metastases(yes/no), ovarian involvement(unilateral or bilateral), and residual disease post ovarian metastasectomy(yes/no). Gastrectomy was defined as gastrectomy concurrent, before or after ovarian metastasectomy; extra-ovarian metastases were determined by CT/MRI tomography or ultrasonography before ovarian metastasectomy; ascites was determined by CT/MRI tomography or ultrasonography before ovarian metastasectomy. Ovarian metastases were diagnosed by pathological examinations after ovarian metastasectomy.

**Table 1:** Correlation between clinicopathological factors and survival of the 133 patients.

Characteristics		No. of patients	mOS (months)	aOS (months)	P value
Age	≤50 year	105	16	22.08	0.054
	>50 year	28	9	13.90	
Ovarian metastases	Metachronous	64	17	22.90	0.087
	Synchronous	69	13	16.51	
Gastrectomy	Yes	89	19	24.93	0.000
	No	44	9	11.56	
Extra-ovarian metastasis	No	119	16	20.38	0.884
	Yes	14	16	17.07	
Ascites	No	49	21	29.86	0.000
	Yes	84	13	13.94	
Ovarian involvement	Unilateral	29	19	22.12	0.185
	Bilateral	104	15	18.65	
Residual disease	No	50	20	28.75	0.000
	Yes	83	13	14.81	

mOS: median overall survival.

aOS: average overall survival.

### Evaluation of Pathological Features and Detection of CSCs Related Molecules Expression in Ovarian Metastastic Lesions

Pathologic type and differentiation, and tumor/stromal cell ratio of each tissue in paraffin sections of ovarian metastases were evaluated by two independent pathologists. The expression of proliferation cell nuclear antigen (PCNA), which is a marker of cells proliferation, in cancer cells of ovarian metastases was detected by immunohistochemical analysis (IHC) and interpreted by two independent pathologists.

The expression of CSCs-related molecules, including CD44, CD133, Oct4, Sox2, p-AKT, p-ERK, Bmi-1, Mel-18 and CBX7, in paraffin sections of ovarian metastases was detected by IHC method. IHC was performed by using a highly sensitive streptavidin-biotin-peroxidase detection system as described [17]. The following primary antibodies were used: anti CD44 (dilution 1∶100; CST), anti CD133 (dilution 1∶100; Miltenyi), anti Oct4 (dilution 1∶100; Abcam), anti Sox2 (dilution 1∶100; CST), anti p-AKT (dilution 1∶100; CST), anti p-ERK (dilution 1∶100; Santa), anti Bmi-1 (dilution 1∶100; CST), anti CBX7 (dilution 1∶100; Abcam), anti Mel-18 (dilution 1∶100; Santa). All slides were interpreted by two independent observers in a blinded fashion. More than 5% of the cells were stained with moderate or strong staining intensity was considered positive, otherwise, the sample was considered negative.

### Ethics Statement

The study protocol was approved by the ethics review board of Shanghai Cancer Center, Fudan University. Written informed consent was obtained from all study participants. All of the procedures were done in accordance with the Declaration of Helsinki and relevant policies in China.

### Statistical Analysis

Overall survival was calculated from the date of ovarian metastasectomy to death or last follow up time. The Kaplan-Meier method was used to calculate survival and log-rank test was performed to compare the difference of overall survival between groups. Independent prognostic factors were determined by multivariate analysis using the Cox proportional hazards model. Pearson χ^2^ test was used to determine the correlation between the expressions of CSCs related molecules in ovarian metastatic specimens and clinicopathologic factors. A value of *P*<0.05 was considered statistically significant. Statistical calculations were performed using SPSS Software Version 15.0 (SPSS Inc.,Chicago, IL,USA).

## Results

### Overall Survival of the 133 Patients and Prognostic Clinical and Pathological Factors

**Figure 1:**
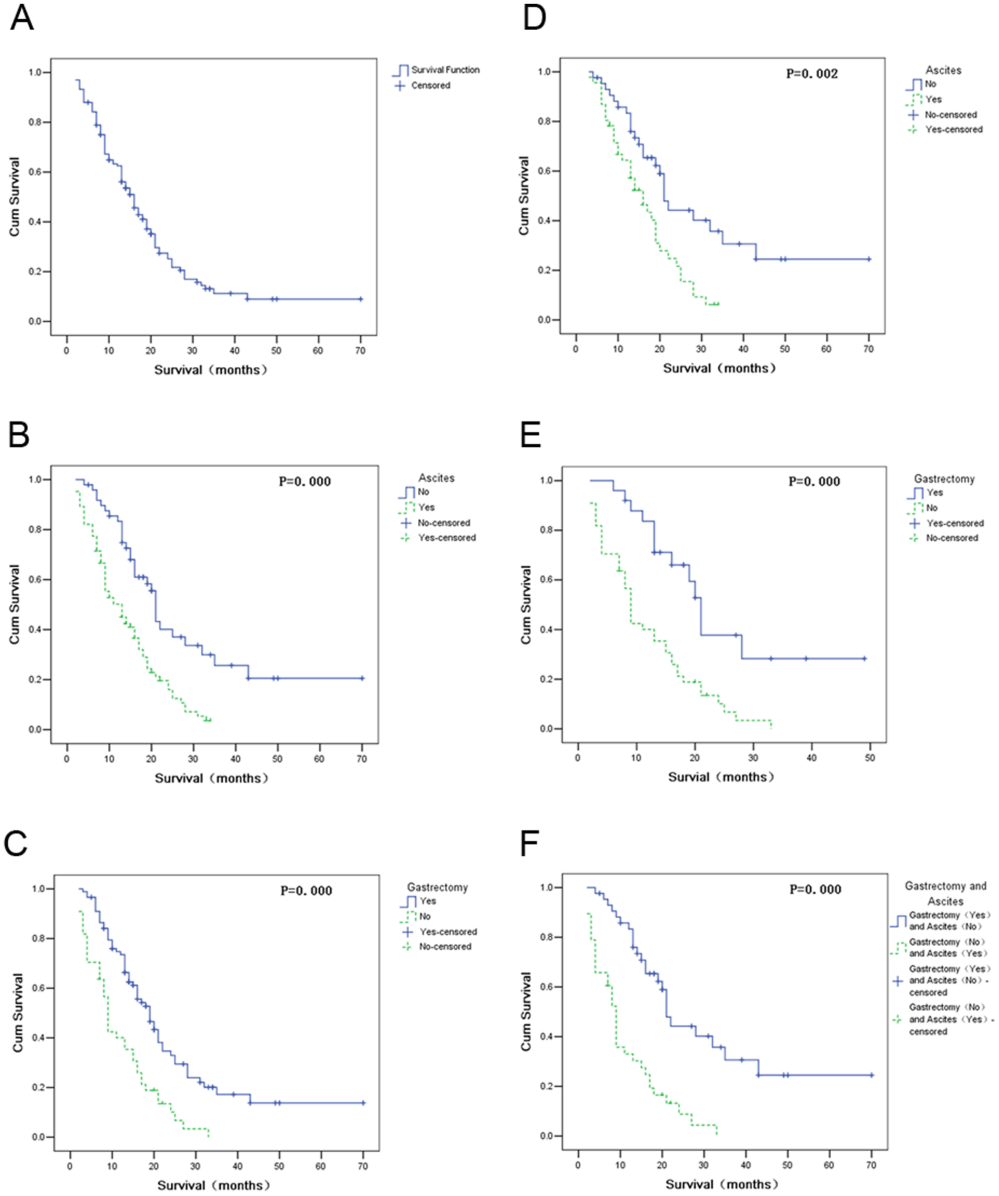
Survival curves of the whole population and subgroups according to prognostic factors. (A) Overall survival curve of the total 133 patients. (B) Survival curves of the patients with or without ascites. (C) Survival curves of the patients who had or hadn’t undergone gastrectomy. (D) Survival curves of the patients with or without ascites in the subgroup of 89 patients who underwent gastrectomy. (E) Survival curves of the patients had or hadn’t undergone gastrectomy in the subgroup of 69 patients with synchronous ovarian metastasis. (F) Survival curves of patients who underwent gastrectomy and without ascites or patients who hadn’t undergone gastrectomy and with ascites.

The overall survival of the 133 patients ranged from 2 to 70 months. The median overall survival(mOS) was 16 months(95%CI:13.33–18.67 months) (Figure 1A), and average overall survival was 20 months(95%CI : 16.78–23.98 months). Univariate analysis showed that no gastrectomy, ascites and residual disease were risk factors correlated with poor survival (Table 1). Multivariate analysis by Cox proportional hazards model showed that ascites (*p = 0.008*, HR 1.917, 95%CI 1.190–3.090) and no gastrectomy (*p = 0.048*, RR 1.598, 95%CI 1.003–2.545) were two independent risk factors associated with poor survival. The mOS of the patients with or without ascites was 13 months (95%CI 8.79–17.21 months) and 21 months (95%CI 19.73–22.27 months) respectively, and the survival of the former was inferior to that of the latter (*p = 0.000*) (Figure 1B). The mOS of the patients who had or had not undergone gastrectomy was 19 months (95%CI 15.35–22.65 months) and 9 months (95%CI 7.96–10.04 months) respectively, and the survival of the former was longer than that of the latter (*p = 0.000*) (Figure 1C). In contrast, age, synchronous or metachronous ovarian metastases, residual diseases post ovarian metastasectomy were not independent prognostic factors by multivariate analysis.

### Subgroup Analysis According to Prognostic Factors

By subgroup analysis, we investigated the prognosis factors in 89 patients who underwent both gastrectomy and ovarian metastasectomy. Among them, 23 patients underwent gastrectomy concurrently with metastasectomy, 64 patients underwent gastrectomy before and 2 after ovarian metastasectomy. Multivariate analysis showed that ascites was an independent risk factor associated with poor survival. The mOS of patients with or without ascites was 16 months (95%CI 18.69–23.31 months) and 21 months (95%CI 11.60–20.40 months) respectively. The survival of patients without ascites was superior to that of the patients with ascites (*p = 0.002*) (Figure 1D).

We also analyzed the 69 patients with synchronous ovarian metastases, including 25 patients underwent both gastrectomy and metastasectomy, and 44 patients didn’t undergo gastrectomy. We found that the survival of the 25 patients who had undergone both gastrectomy and metastasectomy was longer than the other 44 patients underwent metastasectomy only (21 *VS.* 9 months, *p = 0.000*) (Figure 1E).

Additionally, the mOS of the patients who had undergone gastrectomy and without ascites was 21 months, while the patients who hadn’t undergone gastrectomy and with ascites was only 9 months. The survival of the former was significantly longer than that of the latter (*p = 0.000*) (Figure 1F).

### Pathological Features and Expression of CSCs Related Molecules in 64 Ovarian Metastatic Specimens

Pathologic type and differentiation evaluation showed that in 64 ovarian metastatic specimens, only one case was middle differentiated adenocarcinoma, other 63 cases were low differentiated, including32 adenocarcinoma, 1 mucinous adenocarcinoma, and 30 signet ring cell carcinoma. The tumor/stromal cell ratio in metastatic specimens ranged from 10% to 85% (median, 35%) and PCNA positive rate of cancer cells ranged from 1% to 90% (median, 60%).

According to the results of the IHC assay of 64 tumor specimens, CD44 and CD133 were located in the cytomembrane with a positive rate of 72% (46/64) and 70% (45/64),respectively; Oct4 was expressed in the nucleus and the positive rate was 79.7% (51/64); Sox2 was distributed in the nucleus with a positive rate of 81.3% (52/64); the major part of p-AKT was located in cytoplasm with a minor distribution in the nucleus, and the positive rate was 93.8% (60/64); p-ERK was majorly distributed in the nucleus with a minor part in cytoplasm, and the positive rate was 64% (41/64). Bmi-1, Mel-18, and CBX7 were majorly located in the nucleus, and the positive rate was 80% (51/64), 37.5% (24/64), and 53.1% (34/64), respectively (Table 2).

**Table 2:** Correlation between expressions of CSCs related proteins and survival.

Factors		No. ofpatients	mOS(months)	aOS(months)	P value
CD44	Positive	46	11	15.57	0.006
	Negative	18	*	44.96	
CD133	Positive	45	9	15.60	0.007
	Negative	19	25	36.75	
OCT4	Positive	51	14	23.83	0.983
	Negative	13	13	19.85	
SOX2	Positive	52	11	15.85	0.001
	Negative	12	*	59.24	
p-AKT	Positive	60	14	23.32	0.821
	Negative	4	3	12.25	
p-ERK	Positive	41	13	19.11	0.653
	Negative	23	17	29.36	
Bmi-1	Positive	51	12	22.18	0.240
	Negative	13	27	20.44	
Mel-18	Positive	24	13	22.29	0.710
	Negative	40	16	19.90	
CBX-7	Positive	34	10	22.88	0.278
	Negative	30	16	21.67	

* More than half of the patients were still alive at last follow up and mOS could not be calculated.

mOS: median overall survival.

aOS: average overall survival.

### The Correlation between CSCs Related Proteins Expression and Clinicopathologic Factors and Survival

**Figure 2:**
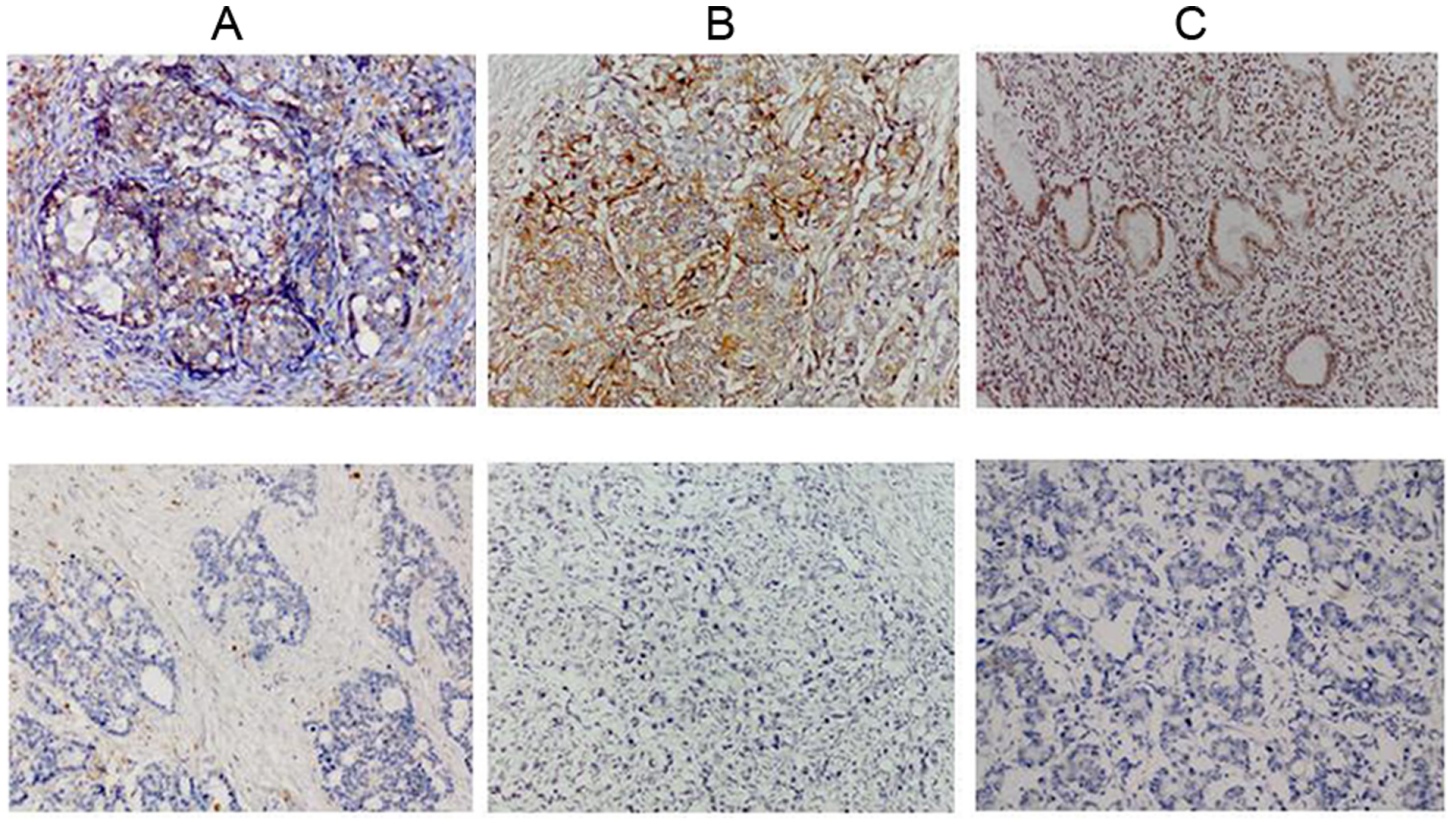
Expressions of three CSCs related molecules in paraffin sections of ovarian metastases. A representative figure of positive (upper panel) or negative (lower panel) expression of (A) CD44, (B) CD133, and (C) Sox2 detected by IHC as described in experimental procedures (original magnification,×200).

**Figure 3:**
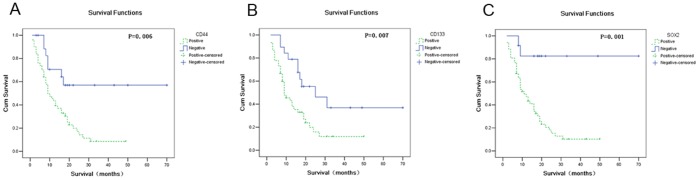
Expressions of three CSCs related molecules in ovarian metastases correlated with patients’ survival. (A) Survival curves of patients with positive or negative expression of CD44. (B) Survival curves of patients with positive or negative expression of CD133. (C) Survival curves of patients with positive or negative expression of SOX2.

In 64 paraffin-embedded ovarian metastatic samples, we examined the association between CSCs related proteins and the survival of patients. Univariate analysis showed that positive expression of CD44, CD133 and Sox2 correlated with poor survival (the positive/negative expression profiles of the three molecules were shown in Figure 2). The average OS in CD44 positive and negative patients was 15.77 months and 44.96 months, respectively (*P = 0.006*) (Table 2, Figure 3A); the average OS in CD133 positive and negative patients was 15.60 months and 36.75 months, respectively (*P = 0.007*) (Table 2, Figure 3B); the average OS in Sox2 positive and negative patients was 15.85 months and 59.24 months, respectively (*P = 0.001*) (Table 2, Figure 3C). These results indicated that CD44, CD133 and Sox2 were risk factors associated with poor survival after ovarian metastasectomy. However, other CSCs related proteins including Oct4, p-AKT, p-ERK, Bmi-1, Mel-18 and CBX7 were not related to prognosis (*P* values ranged from 0.240 to 0.983) (Table 2).

In these 64 cases, age, synchronous or metachronous ovarian metastases, gastrectomy, ascites, and residual disease were also correlated with survival by univariate analysis. Other clinicopathologic factors, including pathologic type, tumor/stromal cell ratio, and PCNA positive rate, were not correlated with survival. Multivariate Cox proportional hazards model analysis, which included CD44, CD133, Sox2, age, synchronous or metachronous ovarian metastases, gastrectomy, ascites, and residual disease, showed that only Sox2 expression was an independent prognostic indicator of overall survival (*p = 0.04*).

We also evaluated the correlation between the expression of CD44, CD133, or Sox2, and clinicopathologic factors including pathologic type, tumor/stromal cell ratio, and PCNA positive rate. We found that there was only a trend of correlation between Sox2 expression and synchronous or metachronous ovarian metastases (*p = 0.071*), or residual disease (*p = 0.087*); CD44 expression and extra-ovarian metastases (*p = 0.077*) (Table 3).

**Table 3:** Correlation between CSCs related proteins expressions and clinicopathological characteristics.

		Sox2			CD44				CD133		
	(+)	(−)	P		(+)	(−)	P		(+)	(−)	P
pathological type											
adenocarcinoma	27	6	0.904		24	9	0.876		24	9	0.663
signet ring cell carcinoma/mucinous adenocarcinoma	25	6			22	9			21	10	
tumor/stromal cell ratio											
>30%	27	7	0.688		25	9	0.754		24	10	0.959
≤30%	25	5			21	9			21	9	
											
PCNA											
>25%	37	10	0.638		33	14	0.945		32	15	0.937
≤25%	11	2			9	4			9	4	
age											
>50 years	10	5	0.202		9	6	0.400		10	5	0.976
≤50 years	42	7			37	12			35	14	
ovarian metastases											
metachronous	28	3	0.071		23	8	0.689		22	9	0.911
Synchronous	24	9			23	10			23	10	
gastrectomy											
No	22	3	0.426		18	7	0.986		20	5	0.174
Yes	30	9			28	11			25	14	
extra-ovarian metastasis											
No	42	12	0.225		36	18	0.077		38	16	1.000
Yes	10	0			10	0			7	3	
ascites											
No	14	6	0.227		12	8	0.154		13	7	0.531
Yes	38	6			34	10			32	12	
ovarian involvement											
Bilateral	41	10	1.000		38	13	0.560		34	17	0.355
Unilateral	11	2			8	5			11	2	
residual disease											
No	18	8	0.087		19	7	0.860		19	7	0.689
Yes	34	4			27	11			26	12	

## Discussion

Previous studies showed that the prognosis of patients with metastatic gastric cancer was very poor and the mOS ranged from 9–11 months after receiving chemotherapy [26]–[28]. Even when Trastuzumab was administered to combine with chemotherapy for advanced or metastatic gastric cancer patients with overexpression of HER-2, mOS was only modestly improved to 13 months [29]. However, the positive rate of HER-2 was low (about 10%–20%), thus only a few patients can get benefit from Trastuzumab administration [30]. In our study, the mOS of the patients underwent ovarian metastasectomy originated from stomach was 16 months, significantly longer than that of patients with metastatic gastric cancer received chemotherapy in previous reports [26]–[29].Thus the outcome possibly favors the role of ovarian metastasectomy in female gastric cancer patients with ovarian metastasis.

Prognostic factors and subgroup analysis can help to select patients who might get benefit from surgical treatment. In the present study, we found that no gastrectomy and with ascites were two independent risking factors associated with poor survival in patients underwent ovarian metastasectomy. The mOS of patients who had undergone gastrectomy was significantly longer than that of patients who had not (19 *vs.* 9 months, *p = 0.048*). Moreover, in the 69 patients with synchronous ovarian metastases, the survival of 25 patients who underwent both gastrectomy and ovarian metastasectomy was also significantly longer than that of the rest 44 patients who hadn’t undergone gastrectomy (mOS: 21 *vs.* 9 months, *p = 0.000*). Some of previous studies indicated that synchronous ovarian metastasis was an unfavorable factor correlated with poor survival [31]. However, in our study, we found that no gastrectomy, but not synchronous or metachronous metastasis, was an independent prognostic factor associated with poor survival. It suggested that gastrectomy or not is more important than synchronous or metachronous metastasis for prognosis. Even in synchronous metastasis, patients underwent both gastrectomy and ovarian metastasectomy survived longer and got benefit from surgical treatment when primary gastric cancer lesion can also been resected. While the mOS of the patients who hadn’t undergone gastrectomy was only 9 months, similar to that of patients with metastatic gastric cancer after receiving chemotherapy [26]–[29]. So survival could not be improved if the original tumor lesion was not eradicated, even though the ovarian metastasis was resected. Due to this finding, we don’t advocate ovarian metastasectomy if the primary site hadn’t or couldn’t been resected.

The survival of patients without ascites was significantly longer than that of patients with ascites (mOS: 21 *vs.* 13 months, *p = 0.008*). Additionally, based on the analysis of the 89 patients who underwent both ovarian metastasectomy and gastrectomy, we also found that ascites was an independent risking factor associated with poor survival *(p = 0.003*). The results were consistent with that in Li’s study [31]. In gastric cancer patients, ascites may be caused by tumor invasion of peritoneum or malnutrition and ascites were usually associated with disseminative metastases in abdomen-pelvic cavities. In the present study, we excluded the patients with low blood albumin in order to exclude the nutritional factor related ascites, so in our study ascites might serve as an indicator of disseminative abdomen-pelvic metastasis. Previous studies have indicated that disseminative abdomen-pelvic metastases correlated with poor prognosis [7]–[9], but it’s more convenient and easier to use ultrasonography or CT/MRI tomography to detect ascites than to detect abdomen-pelvic metastases especially in those patients with disseminative but small metastatic tumor lesions. So it was rational for us to select ascites as the parameter to predict patients’ outcome prior to ovarian metastasectomy. We did not find residual disease or unilateral ovarian metastases as risk factors associated with survival, which were proposed in other studies [7]–[10]. We speculated that, this may be related to the retrospectively design of our study and confounding factors.

As the mOS of the patients who had undergone gastrectomy and without ascites was 21 months, which is much longer than that of the patients with metastatic gastric cancer receiving chemotherapy (9–11 months) in previous reports, it is rational to conclude that this subgroup of patients could get benefit from and be potential candidate for surgical treatment. On the other hand, as the mOS of the patients with primary lesion hadn’t been resected or with ascites was similar with that of the patients with metastatic gastric cancer receiving chemotherapy in literature, ovarian metastasectomy should not be recommended for these patients.

There was significant correlation between the expression of three CSCs related molecules (Sox2, CD44 and CD133) in ovarian metastases and the survival of patients. CD44 and CD133 were extensively investigated and regarded as CSCs markers in a series of tumors including gastrointestinal tumors [20], [21]. CD44 has numerous functions, such as supporting cell migration and transmitting proliferation signals [19]. CD133 was widely distributed in gastric cancer cells and when it was antagonized by special antibody, the growth of gastric tumor will be inhibited [32]. Futhermore, it was reported that CD133 was an independent prognostic factor superior to the depth of invasion and similar to nodal involvement in gastric cancer [33]. In the present study, among 64 cases, the positive rates of CD44 and CD133 in ovarian metastatic specimens were 71.9% and 70.3%, respectively. The survival of patients with positive expression of CD44 or CD133 in tumor tissue was much shorter than that of patients with negative expression. These results suggested that CD44 and CD133 were risk factors correlated with poor prognosis.

Sox2 is a key transcription factor required for maintaining cells’ pluripotency. Overexpression of Sox2, Oct4, Klf4 and c-Myc in somatic/mature cells could generate induced pluripotent stem cells (iPS), showing the critical role of Sox2 and Oct4 in maintaining the pluripotency of stem cells [34]. Sox2 plays an important role in the regulation of organ development and cell type specification, especially in the development of embryonic stem cells (ESCs) [35]. Sox2, or Oct4 also plays a pivotal role in cancer development [36], [37]. In the present study, we found that Sox2, but not Oct4, was a risk factor associated with poor survival. Importantly, Sox2 expression was an independent prognostic indicator of overall survival, which suggests its’ importance in determining the prognosis and that it might serve as a prognostic indicator for predicting patients’ survival and be helpful for the selection of patients in future.

Though PI3K/AKT and ERK-MAPK signaling pathways were reported to be essential for maintaining the pluripotency of stem cells and play important roles in cancer progress [23], [38], we didn’t find p-AKT and p-ERK, activated molecules of these two pathways, correlated with prognosis according to our present results. We also didn’t find that the expression of Bmi-1, Mel-18 and CBX7, which are the members of polycomb family proteins, correlated with the prognosis. Polycomb group (PcG) proteins play important roles in the development of vertebrate organisms, regulation of cell proliferation, senescence and tumorigenesis. We have found that Bmi-1 and CBX7 were overexpressed in gastric cancer tissues and correlated with cancer progress and prognosis, while Mel-18 expression inversely correlated with Bmi-1 expression and Mel-18 could negatively regulate Bmi-1 [39]–[41]. Although we didn’t find Bmi-1, Mel-18 or CBX-7 expression were prognostic factors, it’s still too early to deny the prognostic value of these proteins as the limited samples in our study.

It’s still uncertain of the existence and the role of CSCs in gastric cancer. The results of high positive expression rate of CSCs related proteins in ovarian metastatic tissues of gastric cancer, and the big difference of mOS between patients with positive and negative expression of some CSCs related molecules (CD44, CD133, or SOX2) suggested the existence of CSCs in gastric cancer and that some CSCs markers or related proteins might serve as prognostic factors and help to select patients who might get benefit from surgical treatment in the future. Due to the retrospective design of our study and the fact that the IHC examination was performed in limited cases, prospective trials are needed to extensively elucidate the factors correlated with survival of patients with ovarian metastases originated from stomach underwent ovarian metastasectomy.

### Conclusions

Ovarian metastasectomy might be helpful for prolonging the survivor time of some patients with Krukenberg tumor originated from stomach. Patients without ascites, and with resected primary gastric cancer lesion could get benefit from and be potential candidate for surgical treatment. We do not recommend patients to undergo ovarian metastasectomy if the primary stomach lesion hadn’t or couldn’t been resected, or ascites was detected. Positive expression of three CSCs related molecules, CD44, CD133 or Sox2, correlated with poor survival, and expression of Sox2 was an independent prognostic factor. Prospective studies are needed to further confirm the benefit of ovarian metastasectomy in selected patients.
